# Blocking lncRNA-SNHG16 sensitizes gastric cancer cells to 5-Fu through targeting the miR-506-3p-PTBP1-mediated glucose metabolism

**DOI:** 10.1186/s40170-022-00293-w

**Published:** 2022-11-29

**Authors:** Yan Ding, Sujie Gao, Jiabin Zheng, Xuebo Chen

**Affiliations:** 1 Department of Cellular and Molecular Biology, School of Science and Engineering, Tulane University, New Orleans, LA 70118 USA; 2 Department of Anesthesia, China-Japan Union Hospital of Jilin University, Changchun, Jilin Province 130033 P.R. China; 3 Department of General Surgery, China-Japan Union Hospital of Jilin University, Changchun, Jilin Province 130033 P.R. China

**Keywords:** lncRNA SNHG16, PTBP1, Warburg effect, Glycolysis, 5-Fu resistance

## Abstract

**Background:**

Gastric cancer (GC) is a commonly occurring human malignancy. The 5-fluorouracil (5-Fu) is a first-line anti-gastric cancer agent. However, a large number of GC patients developed 5-Fu resistance. Currently, the roles and molecular mechanisms of the lncRNA-SNHG16-modulated 5-Fu resistance in gastric cancer remain elusive.

**Methods:**

Expressions of lncRNA, miRNA, and mRNA were detected by qRT-PCR and Western blot. RNA-RNA interaction was examined by RNA pull-down and luciferase assay. Cell viability and apoptosis rate under 5-Fu treatments were determined by MTT assay and Annexin V assay. The glycolysis rate of GC cells was evaluated by glucose uptake and ECAR.

**Results:**

Here, we report that SNHG16 as well as PTBP1, which is an RNA-binding protein, are positively associated with 5-Fu resistance to gastric cancer. SNHG16 and PTBP1 were significantly upregulated in gastric tumors and cell lines. Silencing SNHG16 or PTBP1 effectively sensitized GC cells to 5-Fu. Furthermore, glucose metabolism was remarkedly elevated in 5-Fu-resistant GC cells. Under low glucose supply, 5-Fu-resistant cells displayed higher vulnerability than parental GC cells. Bioinformatic analysis and luciferase assay demonstrated that SNHG16 downregulated miR-506-3p by sponging it to form a ceRNA network. We identified PTBP1 as a direct target of miR-506-3p in GC cells. RNA-seq results unveiled that PTBP1 positively regulated expressions of multiple glycolysis enzymes, including GLUT1, HK2, and LDHA. Bioinformatic analysis illustrated the 3′UTRs of glycolysis enzymes contained multiple PTBP1 binding sites, which were further verified by RNA pull-down and RNA immunoprecipitation assays. Consequently, we demonstrated that PTBP1 upregulated the mRNAs of glycolysis enzymes via promoting their mRNA stabilities. Finally, in vivo xenograft experiments validated that blocking the SNHG16-mediated miR-506-3p-PTBP1 axis effectively limited 5-Fu-resistant GC cell originated-xenograft tumor growth under 5-Fu treatments.

**Conclusions:**

Our study demonstrates molecular mechanisms of the SNHG16-mediated 5-Fu resistance of GC cells through modulating the miR-506-3p-PTBP1-glucose metabolism axis, presenting a promising approach for anti-chemoresistance therapy.

**Supplementary Information:**

The online version contains supplementary material available at 10.1186/s40170-022-00293-w.

## Introduction

Gastric cancer (GC), one of the most common malignancies, is the second leading cause of human mortality due to its tumorigenesis, development, and metastasis [1, 2]. Although surgical and chemotherapeutic approaches have improved, since most GC patients are diagnosed in advanced and/or metastatic stages, the overall prognosis and survival rates of GC patients are disappointing [3]. 5-Fluorouracil (5-Fu) is a first-line chemotherapeutic agent against GC by blocking the DNA synthesis of tumor cells via inhibiting the thymidylate synthase activity [4]. However, the development of 5-Fu resistance arose as a major obstacle for 5-Fu-based chemotherapy [5]. Thus, it is urgent to investigate the underlying molecular mechanisms of 5-Fu resistance and develop effectively anti-chemoresistant strategies for 5-Fu-resistant GC patients.

Long non-coding RNAs (lncRNAs), a novel class of noncoding RNAs, are endogenous RNAs with relatively large sizes (exceeding 200 nucleotides) [6]. Accumulating studies revealed that lncRNAs play vital roles in diverse tumor progressions including tumorigenesis, migration, metabolism, and chemoresistance [7]. LncRNA SNHG16 (small nucleolar RNA host gene 16) is a member of the small nucleolar RNA host gene family which is highly expressed in multiple cancers [8]. For instance, studies reported that SNHG16 was overexpressed in colon cancer and regulated by the Wnt pathway [9]. In addition, silencing SNHG16 inhibited prostate cancer cell growth [10]. Studies have uncovered a lncRNA-miRNA network where lncRNAs function as competitive endogenous RNAs (ceRNA) of miRNAs to rescue the miRNA-targeted mRNA genes expressions [11], suggesting the lncRNA-miRNA network is a potential target for anti-cancer therapy. Currently, the precise biological roles and molecular targets of SNHG16 in 5-Fu-resistant gastric cancer have not been elucidated.

Tumor cells display a propensity that they metabolize glucose anaerobically rather than aerobically, associated with increased lactate production, even under sufficient oxygen supply [12]. This phenomenon is known as the “Warburg Effect” [13]. Moreover, this feather of cancer cells is tightly correlated to chemoresistance [14]. We and other colleagues have reported that 5-Fu-resistant cancer cells displayed upregulated glycolysis and could be re-sensitized by glycolysis inhibition [15], suggesting that reversing the Warburg effect could effectively enhance the therapeutic outcomes of traditionally anti-cancer agents. Polypyrimidine-tract-binding protein (PTBP1) is an RNA-binding protein acting as a splicing factor involved in diverse biological processes [15]. Particularly, PTBP1 has been reported to function as an important regulator in cancers, including glioma [16], breast cancer [17], gastric cancer [18], lung cancer [19], renal cell carcinoma [20], and colon cancer [21]. Studies have proven that PTBP1 has the capacity to promote glycolysis through modulating the pyruvate kinase M2 isoform (PKM2), which is a critical regulator of glycolysis [22, 23]. However, the precise molecular mechanisms for the PTBP1-regulated glycolysis and the roles of PTBP1 in 5-Fu resistance in gastric cancer cells have not yet been elucidated.

In this study, the biological roles of lncRNA SNHG16 and PTBP1 in 5-Fu-resistant gastric cancer will be investigated. The miRNA targets of SNHG16 will be identified. Furthermore, we will assess the mechanisms for the PTBP1-mediated glucose metabolism in gastric cancer. The combined anti-tumor effects of PTBP1 inhibition plus 5-Fu treatments will be verified using in vitro and in vivo models. Our study demonstrates a non-coding RNA-based therapeutic strategy as well as its underlying molecular mechanism, providing promising therapeutic approaches to overcome 5-Fu resistance in gastric cancer.

## Materials and methods

### Patient samples

A total of 55 gastric tumor tissues and matched adjacent normal tissues were evaluated in this study. Tissues were obtained from patients who underwent surgery at the Department of General Surgery, China-Japan Union Hospital of Jilin University, China, between 2015 and 2018. After surgical dissection, patient samples were immediately frozen with liquid nitrogen and stored at −80°C. Patients did not receive chemo- or radio-therapy before surgery. This study was approved by the ethics committee from the Institutional Review Board of the China-Japan Union Hospital of Jilin University, Changchun, Jilin Province, P. R. China. All participants gave written informed consent.

### Cell culture and reagents

Five gastric cancer cell lines (AGS, BGC-823, MKN-45, MGC-803, and SGC-7901) and one normal gastric mucosa endothelial cell line GES-1 were purchased from the Cell Research Institute of the Chinese Academy of Sciences (Shanghai, China). Cells were cultured in RPMI 1640 medium (Thermo Fisher Scientific, Inc., Carlsbad, CA, USA) with 10% fetal bovine serum (FBS) (Thermo Fisher Scientific, Inc., Carlsbad, CA, USA) and 100 units/ml penicillin plus 100 μg/ml streptomycin (Thermo Fisher Scientific, Inc., Carlsbad, CA, USA) at 37°C in a humidified cell culture incubator with 5% CO_2_. The 5-Fu-resistant gastric cancer cell line AGS/5-Fu R was established according to the previous description by continually exposing to increased concentrations of 5-Fu. The acquired 5-Fu-resistant cells were re-selected by treating with 5-Fu at 300 uM every 2 months. The rabbit anti-GLUT1 (#73015), rabbit anti-Hexokinase 2 (#2867), rabbit anti-LDHA (#3582), and rabbit anti-β-actin (#4970) antibodies were purchased from the Cell Signaling Technology (Danvers, MA, USA). Mouse anti-PTBP1 (#32-4800) monoclonal antibody was purchased from Thermo Fisher Scientific (Shanghai, China). 5-Fu, 2-DG, and Oxamate were purchased from Sigma-Aldrich (Shanghai, China).

### Construction and transfections of plasmid DNA, siRNA, and miRNA

The pCDNA3.1-PTBP1 overexpression plasmid was constructed according to the previous description [24]. Fragment encoding the full-length cDNA of PTBP1 was ligated into the pcNDA3.1 vector. The small interfering RNA (siRNA) targeting SNHG16 was designed and synthesized by Sangon Biotech (Shanghai, China). The PTBP1 shRNA was synthesized by ABLife Biotech (Wuhan, China), silence sequence of PTBP1: GCGTGAAGATCCTGTTCAATA. Sense and antisense strands were annealed to be shRNA. Vector pGFP-B-RS was digested by HindIII and BamHI at 37°C for 2h~3h. A linearized vector DNA digested by HindIII and BamHI and insert shRNA were ligase by T4 DNA Ligase (NEB). The interference sequence of shRNA was verified by Sanger sequencing. Control miRNA and miR-506-3p precursor were purchased from GenePharma Co. (Shanghai, China). Transfections were performed using the Lipofectamine 2000 (Invitrogen, California, USA) according to the manufacturer’s instructions. MiRNAs or siRNAs were transfected at 50 nM. Plasmid DNA was transfected at 2 μg. After 48 h, cells were collected for the following experiments.

### RNA isolation and qRT-PCR

The total RNA was isolated from surgically resected gastric tumors or cultured gastric cancer cells using the TRIZOL reagent (Ambion, USA). The RNA was further purified with two phenol-chloroform treatments and then treated with RQ1 DNase (Promega, Madison, WI, USA) to remove DNA. The quality and quantity of RNA samples were assessed by measuring the absorbance at 260 nm/280 nm (A260/A280) using Smartspec Plus (BioRad, Hercules, CA, USA). The first-strand cDNA was synthesized using 0.5 ug of total RNA using a SuperScript First-Standard Synthesis System for RT-PCR (Invitrogen, Carlsbad, CA, USA) following the manufacturer’s protocol. qRT-PCR experiments were performed using the SYBR Green qPCR Master Mix (ThermoFisher Scientific, Shanghai, China). Samples were analyzed using an ABI Prism 7700 Sequence Detection System (Applied Biosystems, Foster City, NJ, USA). Primers for qRT-PCR were as follows: miR-506-3p: Forward: 5′-GCCACCACCATCAGCCATAC-3′, Reverse: 5′-GCACATTACTCTACTCAGAAGGG-3′; SNHG16: Forward: 5′-GCAGAATGCCATGGTTTCCC-3′, Reverse: 5′-GGACAGCTGGCAAGAGACTT-3′; PTBP1: Forward: 5′-GCATCGACTTTTCCAAGCTC-3′, Reverse: 5′-GGAAACCAGCTCCTGCATAC-3′; GLUT1 Forward: 5′-TTGCAGGCTTCTCCAACTGGAC-3′, Reverse: 5′-CAGAACCAGGAGCACAGTGAAG-3′; HK2: Forward: 5′-TACACTCAATGACATCCGAACTG-3′, Reverse: 5′-CGTCCTTATCGTCTTCAATATCC-3′; LDHA: Forward: 5′-ATGAAGGACTTGGCGGATGA-3′, Reverse: 5′-ATCTCGCCCTTGAGTTTGTCTT-3′; β-actin: Forward: 5′-CTGAGAGGGAAATCGTGCGT-3′, and Reverse: 5′-CCACAGGATTCCATACCCAAGA-3′. The expression of β-actin was used to normalize the relative expression levels. Human U6 was an internal control for miRNA detection. The thermal profile was set as follows: 95°C for 1 min and 40 cycles at 95° C for 15 s, 58°C for 20 s, and 72°C for 20 s. Experiments were performed in triplicate and repeated three times. The results were analyzed using the 2^−ΔΔCt^ method.

### RNA-Seq analysis

The quality and quantity of the purified RNA samples were redetermined by measuring the absorbance at 260 nm/280nm (A260/A280) using Smartspec Plus (BioRad, USA). The integrity of RNA was further verified by 1.5% agarose gel electrophoresis. For each sample, 1μg of the total RNA was used for RNA-seq library preparation by VAHTS Stranded mRNA-seq Library Prep Kit (Vazyme Biotech Co., Ltd, Nanjing, China). Polyadenylated mRNAs were purified and fragmented and then converted into double-strand cDNA. After the step of end repair and A tailing, the DNAs were ligated to VAHTS RNA Adapters (Vazyme Biotech Co., Ltd, Nanjing, China). Purified ligation products corresponding to 200–500 bps were digested with heat-labile UDG, and the single-strand cDNA was amplified, purified, quantified and stored at −80°C before sequencing. For high-throughput sequencing, the libraries were prepared following the manufacturer’s instructions and applied to Illumina HiSeq X Ten system for 150 nt paired-end sequencing.

### RNA-Seq raw data clean and alignment

Raw reads containing more than 2-N bases were first discarded. Then, the adaptors and low-quality bases were trimmed from raw sequencing reads using FASTX-Toolkit (Version 0.0.13). The short reads less than 16nt were also dropped. After that, clean reads were aligned to the GRch38 genome by tophat2 [25] allowing 4 mismatches. Uniquely mapped reads were used for gene reads number counting and FPKM calculation (fragments per kilobase of transcript per million fragments mapped).

### Functional enrichment analysis

To sort out functional categories of DEGs, Gene Ontology (GO) terms were identified using KOBAS 2.0 server [26]. Hypergeometric test and Benjamin-Hochberg FDR controlling procedure were used to define the enrichment of each term.

### Differentially expressed gene (DEG) analysis

The R/Bioconductor package edgeR [27] was utilized to screen out the differentially expressed genes (DEGs). A false discovery rate <0.05 and fold change >2 or < 0.5 were set as the cutoff criteria for identifying DEGs.

### Bioinformatic analysis

The interaction between lncRNA-SNHG16 and miR-506-3p was predicted by starBase of ENCORI (http://starbase.sysu.edu.cn/). The correlation between SNHG16 and PTBP1 expressions in gastric cancer patient was analyzed by starBase of ENCORI. The binding of miR-506-3p and PTBP1 was predicted from starBase of ENCORI. Survival rates according to SNHG16, miR-506-3p, and PTBP1 expressions in gastric cancer patients were analyzed by www.kmplot.com. 3′UTRs of glycolysis enzymes were obtained from NCBI. Binding motifs of PTBP1 were analyzed from starBase of ENCORI. *p*<0.05 was considered statistical significance.

### RNA immunoprecipitation (RIP)

RNA was isolated from GC cells using a Rneasy Mini kit (Qiagen, Hilden, Germany). GC cells were lysed in RIP lysis buffer from the Magna RIPTM RNA-binding protein immunoprecipitation kit (Millipore, Bedford, MA, USA). Anti-IgG (ab172730, Abcam) or anti-PTBP1 (#32-4800, Thermofisher) antibody with A/G immunomagnetic beads were premixed in immunoprecipitation buffer. Cell lysates were mixed with above reaction to immuno-precipitate PTBP1-RNA complexes at 4^o^C for 16 h. Proteinase K was then added into the reaction. RNA samples were purified. The enrichments of GLUT1, HK2, and LDHA mRNAs which bond with PTBP1 were analyzed by qPCR and RT-PCR. Experiments were repeated three times.

### RNA pull-down assay

RNA pull-down assay was performed according to previous report. Briefly. 3′UTRs of glycolysis enzymes and the negative control (antisense 3′UTR and scramble) were in vitro transcribed and biotin labeled using a biotin RNA labeling mix (Roche, Shanghai, China). Mixture was treated with RNase-free DNase I and purified by a RNeasy mini kit (Qiagen, Hilden, Germany). Proteins from GC cell extracts were incubated with the above biotinylated RNAs at 50 pmol/mg followed by incubation with streptavidin agarose beads (Invitrogen, Carlsbad, CA, USA). After washing, protein samples were detected by Western blot. Experiments were repeated three times.

### RNA stability assay

GC cells were first transfected with control siRNA or siPTBP1 for 48 hours. Actinomycin D (ActD) was added at a final concentration of 5 μg/ml and incubated for various periods. Total RNA was isolated and transcripts of glycolysis enzymes were examined by qRT-PCR. β-actin was an internal control.

### Seahorse metabolic flux analysis

Extracellular acidification rate (ECAR) and oxygen consumption rate (OCR) were detected using the Seahorse XFp Analyzer (Agilent, Santa Clara, CA, USA) and detection kits from Agilent Technologies Inc. (Santa Clara, CA, USA) according to the manufacturer’s instructions. Equal number of cells from each treatment were analyzed. Results were normalized by protein concentrations. Experiments were performed in triplicate and repeated three times.

### Glucose uptake and lactate production

Gastric cancer cells were seeded on a 12-well plate (1×10^5^ per well). The detections of glucose uptake and lactate production were performed using the Glucose Uptake Colorimetric Assay Kit (Applygen Technologies, Beijing, China) and the l-lactate assay kit (BioVision, Milpitas, CA, USA) according to the manufacturer’s instructions. Results was normalized by the cell number of each well. Relative glycolysis rate was calculated from the absorbance of drug-treated cells/untreated cells. Experiments were performed in triplicate and repeated three times.

### Luciferase assay

Gastric cancer cells (5 × 10^4^ cells/well) were seeded in 24-well plates and cultured for 24 h. Cells were then co-transfected with control miRNA or miR-506-3p plus 50 ng pGL3-reporter luciferase reporter containing WT- or Mut- SNHG16 or PTBP1 3′UTR using Lipofectamine 2000 (Thermo Fisher Scientific Inc., Waltham, MA, USA). Forty-eight hours post-transfection, luciferase activity was measured using a Dual luciferase reporter assay system (Promega, Madison, WI, USA) according to the manufacturer’s instructions. Firefly luciferase activity was normalized to that of the Renilla luciferase. Experiments were performed in triplicate and repeated three times.

### Cell viability assay

Cell viability was examined by MTT (3-(4,5-dimethylthiazol-2-yl)-2,5-diphenyltetrazolium bromide) assay (Sigma-Aldrich, Shanghai, China). Gastric cancer cells (5×10^3^ cells/well) were seeded into 96-well plates at 80% confluence for 24 h. Cells were washed with PBS. Medium was refreshed and MTT solution was added at 37 °C for 2 h. Then, 150 μl of DMSO was added to dissolve the formazan crystals. The optical density (OD) of formazan concentrations was determined at 570 nm using a microplate reader (Bio-Rad Laboratories). The OD values were normalized by cell numbers. Relative viability was obtained from the absorbance at 540 nm of drug-treated cells/the absorbance at 540 nm of untreated cells. Experiments were performed in triplicate and repeated three times.

### Clonogenic assay

Anchorage-dependent GC cells (1 × 10^3^ cells/well) were plated onto 6-well plate for 24 h. After 5-Fu treatment, medium was refreshed, and cells were cultured for additional 10 days then stained by 1% crystal violet solution. The survival clones were examined under microscopy. Experiments were repeated three times.

### Cell apoptosis assay

GC cells (3 × 10^5^ per well) were seeded onto 6-well plates for 24 h. Cell apoptosis rate was assessed by FITC-Annexin V/PI Kit (#556547, BD Biosciences, San Jose, CA, USA) according to the manufacturer’s instructions. Briefly, after treatments, cells were washed twice with cold PBS. Cells were stained with FITC-Annexin V (5 ml) and PI (5 ml) for 30 min at room temperature in dark. Apoptosis was analyzed using a BD Accuri C6 flow cytometer (BD Biosciences, San Jose, CA, USA). Experiments were repeated three times.

### Western blot

Proteins were extracted from cells using the RIPA lysis buffer (Beyotime Ltd., Shanghai, China) suppled with 1 × protease inhibitor cocktail (Sigma-Aldrich, Shanghai, China). Lysates were incubated on ice for 15 min and centrifugated at 10,000 g for 15 min at 4 °C. Protein concentrations were measured by Bradford method. Equal amounts of proteins were applied to a 10% sodium dodecyl sulfate-polyacrylamide gel (SDS-PAGE) and transferred to a nitrocellulose membrane. Membranes were blocked with 5% nonfat milk at room temperature for 1 h. After complete washing by PBST, membranes were incubated with primary antibodies (1:1000 dilution) with gentle shaking at 4°C overnight. After washing, the membranes were incubated with HRP-conjugated goat anti-mouse or goat anti-rabbit antibody (1:3000 dilution) at room temperature for 1 h. The detection of antibody-bound protein signals was performed using the enhanced chemiluminescence kit (Bio-Rad Ltd., USA). Experiments were repeated three times.

### Xenograft mice model

All of the xenograft experiments were complied with the guidelines of the Institutional Animal Care and Use Committee of the China-Japan Union Hospital of Jilin University. Totally, thirty-two 6-week-old nude mice were used in this study. Mice were kept on a regular 12/12-h light-dark cycle cages. Nude mice were separated to two groups (16 each), then were injected subcutaneously with AGS cells (8x10^6^) transfected with control shRNA or PTBP1 shRNA to establish xenograft models. Mice from each xenograft group were randomly divided into two groups and treated as follows: PBS control; 5-Fu alone [40 mg/kg intraperitoneal (i.p.), 2 times/wk]; and sh PTBP1 alone and 5-Fu plus sh PTBP1. Mice mortality was monitored daily. Tumors from dead mice were collected at the death time point. After 80 days, the survival mice were euthanized by CO_2_ method and the xenograft tumor tissues were dissected. Tumors were stored at −80°C for downstream analysis. Experimental protocol was carried out in accordance with the European Communities Council Directive of 24 November 1986 (86/609/EEC) and approved by an institutional review committee from Institutional Animal Care and Use Committee of the China-Japan Union Hospital of Jilin University.

### Statistical analysis

Statistical difference was analyzed using the GraphPad Prism 7.0 software. Results are presented as the mean ± SD. The unpaired Student’s *t* test was used for the data analysis between two groups and significance among three or more groups was analyzed by one-way ANOVA followed by Bonferroni corrections. All experiments were performed in triplicate and repeated three times. A statistical difference of *p* < 0.05 was considered significant.

## Results

### LncRNA-SNHG16 is significantly upregulated in gastric cancer and positively correlated with PTBP1 expression

**Fig. 1:**
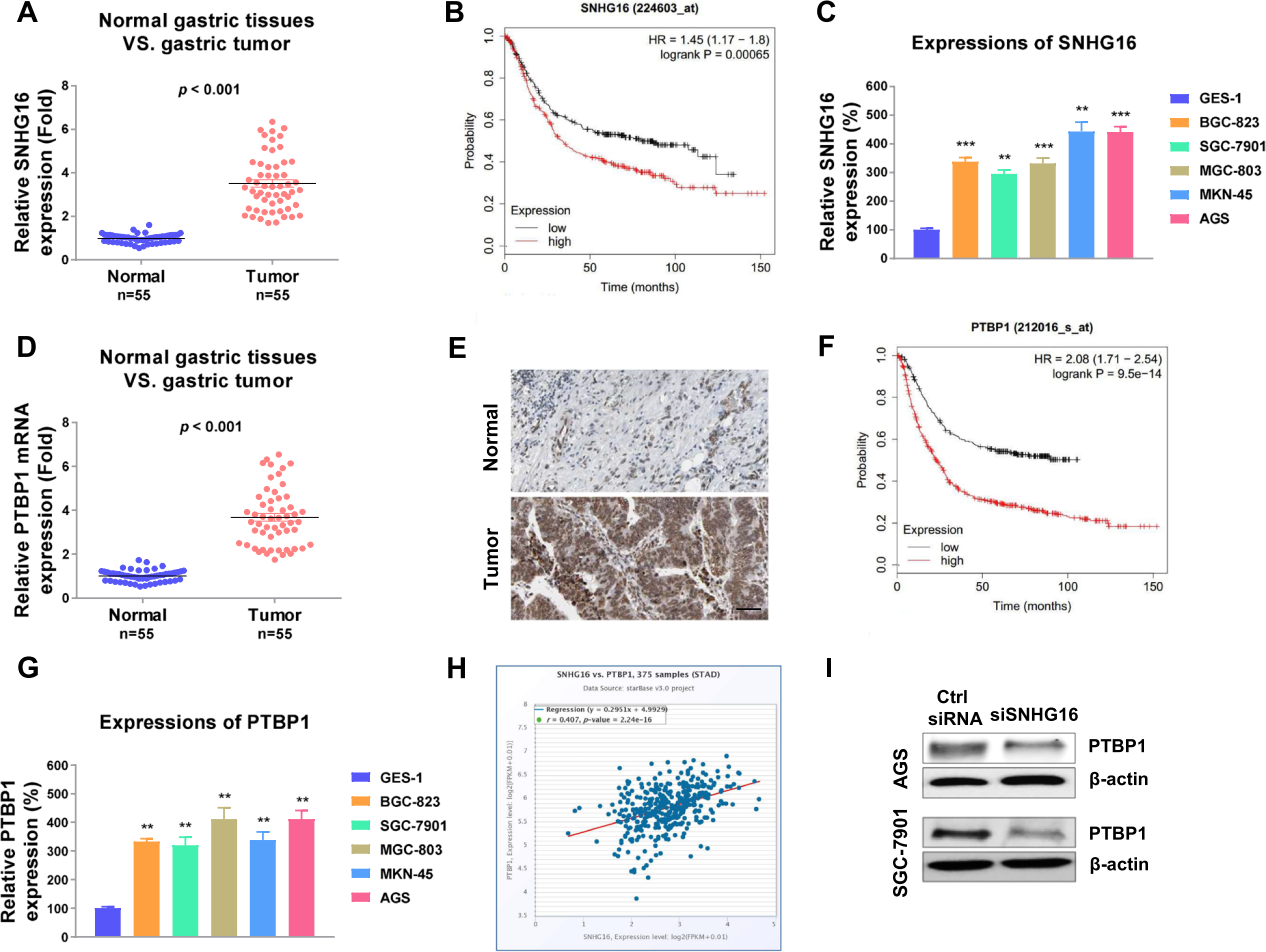
SNHG16 and PTBP1 are positively associated with gastric cancer. **a** SNHG16 expressions were detected in gastric tumors (*n*=55) and their matched normal gastric tissues by qRT-PCR. **b** Kaplan-Meier Plotter analyzes the survival rates of GC patients with high or low SNHG16 expressions. **c** Expressions of SNHG16 were detected in one non-tumorigenic gastric epithelial cell line, GES-1, and five GC cell lines by qRT-PCR. **d** PTBP1 expressions were detected in gastric tumors (*n*=55) and their matched normal gastric tissues by qRT-PCR and **e** IHC. **f** Kaplan-Meier Plotter analyzes the survival rates of GC patients with high or low PTBP1 expressions. **g** Expressions of PTBP1 were detected in one non-tumorigenic gastric epithelial cell line, GES-1, and five GC cell lines by qRT-PCR. **h** Pearson coefficient analysis shows a significantly positive correlation between SNHG16 and PTBP1 in gastric tumors. **i** AGS and SGC-7901 cells were transfected with control or SNHG16 siRNA, and protein expressions of PTBP1 were examined by Western blot. Data were presented as mean±S.D. *p<0.05; **p<0.01; ***p<0.001

Accumulating evidence revealed that lncRNA SNHG16 and PTBP1 were apparently associated with poor prognosis and malignant phenotypes of multiple cancers [8–10, 15], we started to investigate the clinical relevance of SNHG16 and PTBP1 in gastric cancer. Totally, fifty-five gastric tumor tissues and their corresponding adjacent normal tissues were collected and the expressions of SNHG16 and PTBP1 were examined by qRT-PCR. We observed that the SNHG16 expressions were significantly upregulated in gastric tumor tissues compared with normal tissues (Fig. 1a, S1a). Consistently, Kaplan-Meier Plotter survival analysis from the kmplot.com indicated high SNHG16 expressions were significantly associated with pool survival rates of gastric cancer patients (Fig. 1b). Meanwhile, with the comparison of SNHG16 from normal gastric epithelial cells, GES-1 with that from other five gastric cancer cell lines, we detected SNHG16 was remarkedly upregulated in gastric cancer cells (Fig. 1b). These results suggest SNHG16 plays an oncogenic role in gastric cancer. Similarly, PTBP1 mRNA and protein expressions were obviously increased in gastric tumors compared with normal gastric tissues by qRT-PCR and IHC (Fig. 1d, e, S1b). Moreover, high PTBP1 expressions were associated with pool survival rates of gastric cancer patients (Fig. 1f). Consistent results from Fig. 1g showed that PTBP1 was significantly upregulated in gastric cancer cells. Intriguingly, bioinformatic analysis from StarBase revealed an apparently positive correlation between SNHG16 and PTBP1 in gastric tumors (Fig. 1h). To assess the regulatory relationship between them, gastric cancer cells, AGS, and SGC-7901 were transfected with SNHG16 siRNA or control siRNA. As we expected, silencing SNHG16 effectively attenuated PTBP1 expressions (Fig. 1i). Taken together, the above results demonstrated lncRNA SNHG16 upregulated PTBP1 in gastric cancer cells and positively associated with gastric tumor progress.

### SNHG16 and PTBP1 promote 5-Fu resistance of gastric cancer cells

**Fig. 2:**
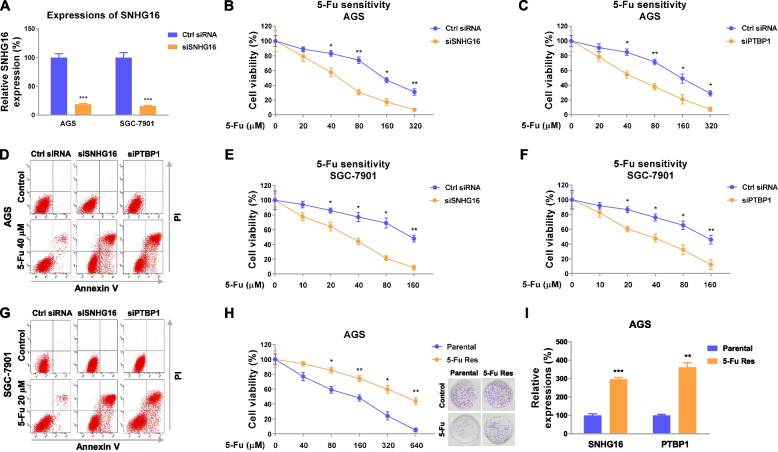
SNHG16 and PTBP1 promote 5-Fu resistance of gastric cancer. **a** AGS and SGC-7901 cells were transfected with control siRNA or SNHG16 siRNA, and expressions of SNHG16 were examined by qRT-PCR. **b**, **c** AGS cells with SNHG16 or PTBP1 silencing were treated with 5-Fu at the indicated concentrations for 48 h. Cell viability was determined by MTT assay and **d** Annexin V apoptosis assay. **e**, **f** SGC-7901 cells with SNHG16 or PTBP1 silencing were treated with 5-Fu at the indicated concentrations for 48 h. Cell viability was determined by MTT assay and **g** Annexin V apoptosis assay. **h** AGS parental cells and 5-Fu-resistant cells were treated with 5-Fu for 48 h. Cell viability was determined by MTT assay and clonogenic assay. **i** Expressions of SNHG16 and PTBP1 were examined in AGS parental and 5-Fu-resistant cells. Data were presented as mean±S.D. *p<0.05; **p<0.01; ***p<0.001

The acquired chemoresistance, which frequently occurs in gastric cancers has attracted global attention. To explore the roles of SNHG16 and PTBP1 in 5-Fu resistance, we assessed the effects of silencing SNHG16 and PTBP1 on 5-Fu sensitivity of gastric cancer cell. SNHG16 was effectively knocked down in AGS and SGC-7901 cells by siRNA (Fig. 2a). AGS and SGC-7901 cells with normal or low SNHG16 expression were treated with elevated concentrations of 5-Fu for 48 h. As we expected, gastric cancer cells with either SNHG16 or PTBP1 silencing displayed increased 5-Fu sensitivity compared with control cells (Fig. 2b, b, and d, S2a). The 5-Fu IC50s of AGS was 138.75 μM. Silencing SNHG16 or PTBP1 effectively dropped IC50s of them to 47.42 or 41.72 μM (Fig. 2b, c, and d). Consistent results were obtained from SGC-7901 cells that knocking down SNHG16 or PTBP1 significantly sensitized GC cells to 5- Fu (Fig. 2e, f, and g, S2b).

To gain an in-depth understanding of the roles of SNHG16 and PTBP1 in 5-Fu-resistant gastric cancer, we established a 5-Fu-resistant gastric cancer cell line from AGS cell by gradual exposing cells to increased concentrations of 5-Fu for 3 months. To verify the 5-Fu resistance, AGS parental and resistant cells were treated with 5-Fu at 0, 40, 80, 160, 320, or 640 μM. As shown in Fig. 2g, parental AGS cells, which have a 5-Fu IC50 at 134.22 μM, showed reduced cell viabilities with 5-Fu treatments. The 5-Fu-resistant AGS cells could tolerate higher concentrations of 5-Fu treatments (IC50: 463.12 μM) (Fig. 2g). Expectedly, SNHG16 and PTBP1 were significantly upregulated in 5-Fu-resistant cells (Fig. 2h, i). The upregulations of SNHG16 and PTBP1 were verified in another 5-Fu-resistant GC cell line, SGC-7901 5-Fu Res (Fig. S3a). In summary, these results demonstrated SNHG16 and PTBP1 are positively associated with 5-Fu resistance in gastric cancer, suggesting targeting SNHG16 and PTBP1 could contribute to enhancing the cytotoxicity of chemotherapeutic agents.

### Fu-resistant GC cells exhibit upregulated glycolysis rate

**Fig. 3:**
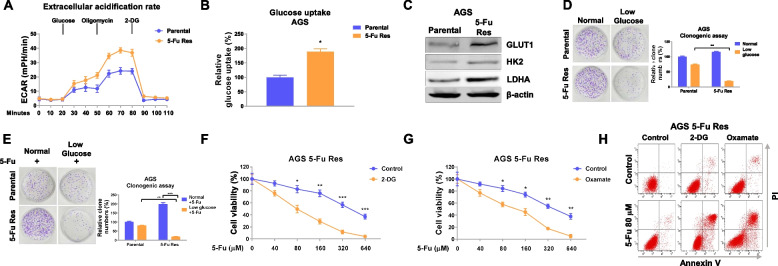
5-Fu-resistant gastric cancer cells display glucose addictive phenotype. **a** ECAR, **b** glucose uptake, and **c** glycolysis enzymes were measured in AGS parental and 5-Fu-resistant cells. d AGS parental and 5-Fu-resistant cells were cultured under normal or low glucose conditions. Cell viability was determined by clonogenic assay. e The above cells under normal or low glucose conditions were treated with 5-Fu for 48 h. Cell viability was determined by clonogenic assay. f, g, h AGS 5-Fu-resistant cells were treated with 5-Fu alone or combined with glycolysis inhibitors at the indicated concentrations. Cell death was determined by MTT assay and Annexin V apoptosis assay. Data were presented as mean±S.D. *p<0.05; **p<0.01; ***p<0.001

Mounting studies revealed that cancer cell displayed dysregulated glucose metabolism which affected chemosensitivity of various anti-cancer drugs [14]. To investigate the mechanisms behind the SNHG16-mediated 5-Fu resistance, we evaluated the glycolytic phenotypes of parental and 5-Fu-resistant gastric cancer cells through Seahorse Extracellular Flux analysis. Dynamically bioenergetic results demonstrated that 5-Fu-resistant cells displayed significantly increased extracellular acidification rate (ECAR) and an overall glucose uptake compared with AGS and SGC-7901 parental cells (Fig. 3a, b, S3b, c). Consistently, expressions of glycolysis key enzymes, GLUT1, hexokinase 2 (HK2), and lactate dehydrogenase-A (LDHA) were significantly upregulated in 5-Fu-resistant gastric cancer cells (Fig. 3c). On the contrary, the mitochondrial respiration rate (OCR) was decreased in 5-Fu-resistant cells (Fig. S4), indicating a reverse “Warburg effect” was observed in 5-Fu-resistant gastric cancer cells. To assess the effects of the elevated glucose metabolism in 5-Fu-resistant cells, AGS parental and 5-Fu-resistant cells were cultured under regular and low glucose medium for 48 h. Under low glucose, in contrast to parental cells which exhibited attenuated growth rate, 5-Fu-resistant cells showed more severe growth inhibition (Fig. 3d). Expectedly, under 5-Fu treatment, 5-Fu-resistant cells displayed significantly increased cell death under low glucose compared with parental cells (Fig. 3e), indicating a glucose-addictive characteristic of 5-Fu-resistant GC cells. To investigate whether targeting the elevated cellular glycolysis rate by glycolysis inhibitors could sensitize 5-Fu-resistant cells, we co-treated AGS 5-Fu-resistant cells with 2-DG or Oxamate, two glycolysis inhibitors with 5-Fu. Results in Fig. 3f, g, and h and S5 clearly demonstrated that blocking glycolysis remarkedly enhanced the 5-Fu sensitivity of AGS 5-Fu-resistant cells. Taken together, these results illustrated targeting glycolysis could be an effective approach to overcome chemoresistance.

### SNHG16 positively associates with glycolysis of GC cells

**Fig. 4:**
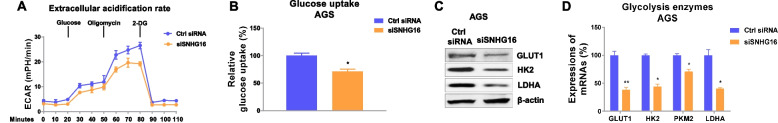
Effects of SNHG16 on glycolysis of GC cells. **a** AGS cells were transfected with control siRNA or SNHG16 siRNA. ECAR, **b** glucose uptake, **c**, **d** glycolysis enzymes were examined. Data were presented as mean±S.D. *p<0.05; **p<0.01

The above results revealed correlations between the SNHG16 and PTBP1-mediated 5-Fu resistance and upregulated cellular glycolysis of 5-Fu-resistant gastric cancer cells. To test whether SNHG16 could regulate glycolysis, the AGS cells were transfected with control siRNA or SNHG16 siRNA. The glycolytic phenotype (ECAR) of SNHG16 silencing cells was significantly decreased by the Seahorse Extracellular Flux analysis (Fig. 4a). In addition, silencing SNHG16 effectively blocked the glucose uptake (Fig. 4b) and glucose metabolism key enzymes, GLUT1, HK2, and LDHA expressions (Fig. 4c, d). Consistently, bioinformatic analysis demonstrated that SNHG16 expressions are positively associated with glycolysis enzyme expressions from the TCGA gastric cancer database (Fig. S6a-c). These results suggest targeting SNHG16 could effectively inhibit the upregulated glucose metabolism of 5-Fu-resistant GC cells.

### miR-506-3p has tumor suppressive roles in gastric cancer and is sponged by SNHG16

**Fig. 5:**
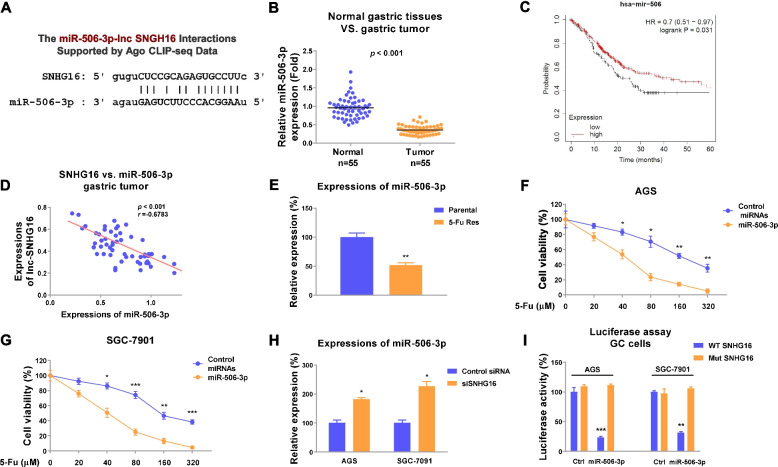
SNHG16 downregulates miR-506-3p in GC by sponging it. **a** The potential binding between SNHG16 and miR-506-3p was predicted from starBase. **b** miR-506-3p expressions were detected in gastric tumors (*n*=55), and their matched normal gastric tissues by qRT-PCR. **c** Kaplan-Meier Plotter analyzes the survival rates of GC patients with high or low miR-506-3p expressions. **d** Pearson coefficient analysis shows a significantly negative correlation between SNHG16 and miR-506-3p in gastric tumors. **e** Expressions of miR-506-3p were detected in AGS parental and 5-Fu-resistant cells. **f**, **g** AGS and SGC-7901 cells were transfected with control miRNA or miR-506-3p. Cells were treated with 5-Fu, and cell viability were determined by MTT assay. **h** GC cells were transfected with control or SNHG16 siRNA. Expressions of miR-506-3p were detected by qRT-PCR. **i** Luciferase assay was performed in AGS and SGC-7901 cells transfected with control or miR-506-3p plus WT-SNHG16 or mut-SNHG16. Data were presented as mean±S.D. *p<0.05; **p<0.01; ***p<0.001

In order to evaluate the mechanisms for the SNHG16-promoted 5-Fu resistance of gastric cancer, we investigated the molecular targets of SNHG16. Studies revealed that lncRNA function as ceRNA of miRNAs through direct sponging them [11]. Bioinformatical analysis predicted that SNHG16 could potentially bind on the seeding region of miR-506-3p (Fig. 5a). Moreover, literature research revealed miR-506-3p was negatively correlated with progresses of multiple cancers [28–30], suggesting miR-506-3p might be a downstream molecule of SNHG16 and involve in 5-Fu resistance. To test this, gastric tumor tissues and their corresponding adjacent normal tissues were collected and the expressions of miR-506-3p were examined by qRT-PCR. Expectedly, miR-506-3p was significantly downregulated in gastric tumor tissues (Fig. 5b). Kaplan-Meier plotter analysis indicated high miR-506-3p expression was associated with a better survival rate of gastric cancer patients (Fig. 5c). Moreover, a significantly invert expression pattern was observed between SNHG16 and miR-506-3p in gastric tumors (Fig. 5d). These results indicated miR-506-3p plays a tumor suppressive role in gastric cancer. To further assess the biological functions of miR-506-3p in 5-Fu resistance, we compared the miR-506-3p expression between AGS parental and 5-Fu-resistant cells. MiR-506-3p was found to be downregulated in 5-Fu-resistant cells (Fig. 5e). In addition, gastric cancer cells with higher miR-506-3p expression exhibited increased 5-Fu sensitivity (Fig. 5f, g), indicating specific delivering miR-506-3p to local tumors could effectively enhance chemotherapy. We then asked whether the SNHG16-miR-506-3p complex forms a ceRNA network in gastric cancer cells. Silencing of SNHG16 apparently increased miR-506-3p expressions in gastric cancer cells (Fig. 5h). To validate the direct binding of SNHG16 on miR-506-3p, a luciferase assay was performed. Results clearly illustrated that transfection of miR-506-3p specifically blocked the luciferase activity of vector containing WT-SNHG16 but not the binding site mutant SNHG16 (Fig. 5i). Collectively, the above results demonstrated miR-506-3p was sponged by SNHG16 and sensitized gastric cancer cells to 5-Fu treatment.

### miR-506-3p inhibits cellular glycolysis through direct targeting PTBP1

**Fig. 6:**
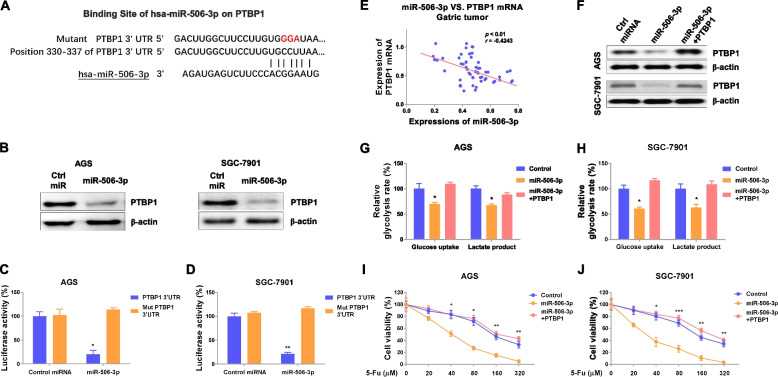
miR-506-3p sensitizes GC cells to 5-Fu by direct targeting PTBP1. **a** The potential binding of miR-506-3p on PTBP1 3′UTR was predicted from starBase. **b** AGS and SGC-7901 cells were transfected with control miRNA or miR-506-3p. Protein expressions of PTBP1 were determined. **c** Luciferase assay was performed in AGS and **d** SGC-7901 cells transfected with control or miR-506-3p plus WT- or mut-3′UTR of PTBP1. **e** Pearson coefficient analysis shows a significantly negative correlation between miR-506-3p and PTBP1 in gastric tumors. **f** AGS and SGC-7901 cells were transfected with control, miR-506-3p alone, or plus PTBP1. Protein expressions of PTBP1 were examined. **g**, **h** The glucose uptake and lactate product from the above transfected cells were determined. **i**, **j** The above transfected cells were treated with 5-Fu at the indicated concentrations, and cell viability was determined by MTT assay. Data were presented as mean±S.D. *p<0.05; **p<0.01; ***p<0.001

Studies reported that microRNAs function through binding to the 3′UTR of target mRNAs [6]. Thus, we analyzed the potential targets of miR-506-3p in gastric cancer. Bioinformatic analysis from the TargetScan.com indicated 3′UTR of human PTBP1 contains putative miR-506-3p binding sites (Fig. 6a). Overexpression of miR-506-3p effectively blocked PTBP1 protein expressions in gastric cancer cells by Western blot (Fig. 6b). To verify whether miR-506-3p could directly bind to the 3′UTR region of PTBP1 mRNA, AGS, and SGC-7901 cells were co-transfected with control miRNAs or miR-506-3p plus WT- or binding site mutant (Mut)- 3′UTR of PTBP1. As we expected, luciferase assay showed exogenous miR-506-3p significantly inhibited the activity of WT-PTBP1 3′UTR but not the Mut PTBP1 3′UTR (Fig. 6c, d). We further detected a significantly reverse correlation between miR-506-3p and PTBP1 mRNA expressions in gastric cancer tissues (Fig. 6e). These results consistently validated PTBP1 could be directly targeted by miR-506-3p in gastric cancer. We next evaluated whether the miR-506-3p-mediated 5-Fu sensitivity was through targeting PTBP1. Consequently, rescue experiments were performed by co-transfection of miR-506-3p plus PTBP1 overexpression plasmid into AGS and SGC-7901 cells (Fig. 6f). Western blot results demonstrated successful rescue of PTBP1 expressions (Fig. 6f). Importantly, restoration of PTBP1 in miR-506-3p overexpressing gastric cancer cells recovered the glucose uptake and lactate product (Fig. 6g, h). Consequently, AGS and SGC-7901 cells with recovery of PTBP1 successfully rescued 5-Fu-resistant phenotypes (Fig. 6i, j). Taken together, the above date demonstrated the miR-506-3p-promoted 5-Fu sensitization of gastric cancer cells was through direct targeting PTBP1.

### PTBP1 promotes glycolysis through regulating glycolysis enzyme expressions

**Fig. 7:**
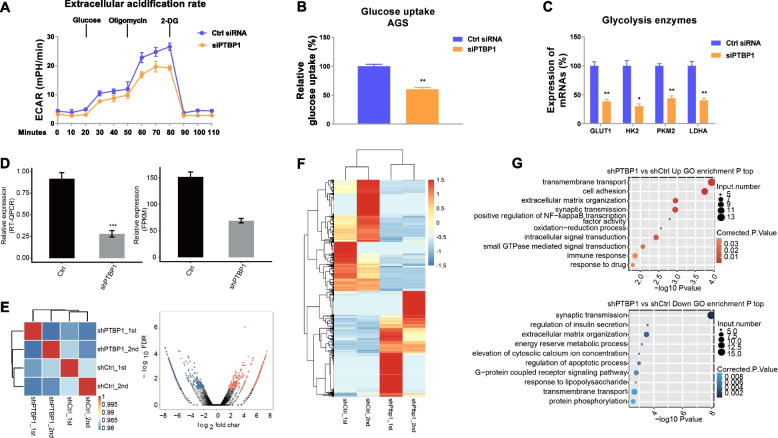
PTBP1 promotes glycolysis of GC cells. **a** ECAR, **b** glucose uptake, and **c** mRNA expressions of glycolysis key enzymes from AGS cells without or with PTBP1 silencing. **d**, **e** Effects of PTBP1 knocking down by shRNA in GC cells. **f** Heatmap from transcriptome sequencing analysis shows differentially expressed mRNAs in AGS parental and 5-Fu-resistant cells. Red: upregulation; Green: downregulation of gene expressions. **g** The top representatively upregulated and downregulated GO enrichment biological processes by shPTBP1. Data were presented as mean±S.D. *p<0.05; **p<0.01

We then asked whether PTBP1 modulates glucose metabolism rate of gastric cancer cells. Bioinformatic analysis from TCGA gastric cancer database illustrated that PTBP1 expressions are positively associated with glycolysis enzymes expressions (Fig. S7a-c). As we expected, AGS cells with PTBP1 silencing showed significantly attenuated ECAR (Fig. 7a), glucose uptake (Fig. 7b), and glycolysis enzyme expressions (Fig. 7c). To further understand the molecular mechanisms for the PTBP1-promoted glycolysis, we performed RNA-Seq experiments by comparison of GC cells with control or PTBP1 silencing (Fig. 7d, e) to explore the transcriptional regulation by PTBP1. Based on the results of RNA-Seq, we analyzed the genes which were differentially expressed in PTBP1-silenced GC cells compared with control GC cells. From the totally 21221 genes, we identified 1067 genes that were regulated by PTBP1 (Fig. 7f). Among them, glycolysis enzymes, GLUT1, HK2, and LDHA were consistently downregulated by PTBP1 silencing (Fig. 7f), suggesting PTBP1 plays critical roles in the transcriptional regulation of glycolysis key enzymes. These regulations were further validated by qRT-PCR from AGS cells by stably knockdown of PTBP1 (Fig. S8). To reveal the potential biological roles of these DEGs (differentially expressed genes), we subjected all 1067 DEGs to GO (Fig. 7g) and KEGG (Fig. S9) annotation. On the base of the cutoff criterion, the upregulated and downregulated genes were respectively enriched in GO terms. In the biological process terms of analysis, the upregulated genes in the PTBP1-KD cells mainly enriched in extracellular matrix organization, transmembrane transport, cell adhesion, synaptic transmission, insulin secretion, and intracellular signal transduction (Fig. 7g, S6). The downregulated genes mostly related to regulation of apoptotic process, G-protein-coupled receptor signal transduction, energy reserve metabolic process, calcium signaling pathway, and synaptic transmission (Fig. 7g, S9). Notably, GO annotation illustrated that PTBP1 silencing negatively affected the metabolic process, consistent with the above-described biological functions of PTBP1 in glucose metabolism of GC cells. Taken together, these results demonstrated that PTBP1 promoted glucose metabolism of gastric cancer cells through upregulating the glycolysis key enzymes.

### PTBP1 stabilizes mRNAs of glycolysis enzymes via direct binding to 3′UTR regions

**Fig. 8:**
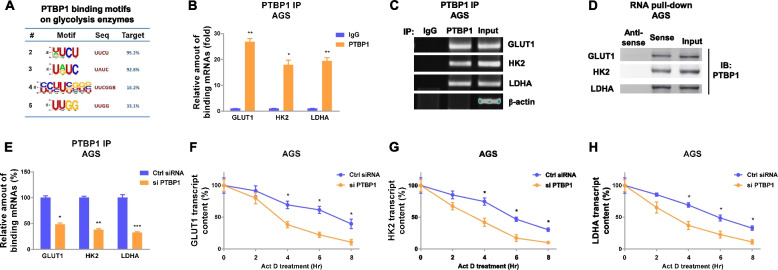
PTBP1 enhances the transcript stability of glycolysis enzymes by binding on 3′UTR regions. **a** Predicted PTBP1 binding motifs on glycolysis key enzymes. **b** RNA immunoprecipitation assay was performed in AGS cells using IgG control or anti-PTBP1 antibody. Glycolysis enzyme mRNA abundance in immunoprecipitated fractions was measured by qRT-PCR and **c** agarose gel electrophoresis. β-actin was an internal control. **d** RNA pull-down assay was performed in AGS cells. Biotin-labeled 3′UTR of glycolysis enzyme, GLUT1, HK2, or LDHA was incubated with cell extracts, respectively. The PTBP1 protein was assayed by western blotting. **e** AGS cells were transfected with control or PTBP1 siRNA, and RNA immunoprecipitation was performed. mRNAs of glycolysis enzymes in immunoprecipitated fraction were determined by qRT-PCR. **f**, **g**, **h** AGS cells were transfected with control or PTBP1 siRNA, followed by Act D treatments. Glycolysis enzyme mRNA stability assays were performed as described in the “Materials and methods” section. Data were presented as mean±S.D. *p<0.05; **p<0.01; ***p<0.001

Previous studies uncovered that PTBP1 functions as an RNA-binding protein to post-transcriptionally regulate target genes expressions [15]. Thus, we analyzed the differentially regulated glycolysis enzymes by PTBP1. Bioinformatics analysis of the PTBP1 targets from starBase predicted that PTBP1 were potentially bond to 3′UTRs of glycolysis enzymes, GLUT1, HK2, and LDHA (Fig. S10a-c). Furthermore, among the predicted multiple PTBP1 binding motifs, we observed that the 3′UTRs of GLUT1, HK2, and LHDA contained conserved PTBP1 binding motifs (Fig. 8a) and the motif #2 was verified to have the strongest binding capacity to 3′UTR of glycolysis enzymes (Fig. S11). Since previous studies described that PTBP1 associates with 3′UTR of target mRNAs to enhance mRNA stability [15], we therefore hypothesized that PTBP1 enhances stabilities of target mRNAs to upregulate glycolysis expressions through directly binding to 3′UTRs of glycolysis enzymes. To evaluate that, RNA immunoprecipitation (RIP) assay was performed in AGS cells. qRT-PCR and RT-PCR results verified that the mRNAs of GLUT1, HK2, and LDHA were apparently enriched in PTBP1-precipitated RNA fragments (Fig. 8b, c). We then performed RNA pull-down assay using biotin-labeled 3′UTRs of glycolysis enzymes. Western blot results in Fig. 8d showed significantly enrichment of PTBP1 which was associated with 3′UTRs of glycolysis enzymes in the protein-RNA complex. To evaluate whether PTBP1 upregulated glycolysis enzyme expressions through binding to their 3′UTRs, AGS cells without or with PTBP1 knockdown were analyzed by RNA immunoprecipitation. Expectedly, AGS cells with PTBP1 silencing showed less amount of glycolysis enzyme mRNA fragments immunoprecipitated by PTBP1 (Fig. 8e). The above results demonstrated specific associations between PTBP1 and 3′UTRs of glycolysis enzymes. To investigate whether PTBP1 affects mRNA stability of glycolysis enzymes, RNA stability assays were carried out to compare the half-lives of GLUT1, HK2, and LDHA mRNAs in control and PTBP1-silencing GC cells. As we expected, the half-lives of glycolysis enzyme mRNAs were significantly cut down in PTBP1-silencing cells compared with those from control cells (Fig. 8f, g, and h). In summary, these results clearly validated that PTBP1 upregulated glycolysis enzyme expressions through direct bind to 3′UTRs of them, resulting in the mRNA stabilization.

### Targeting the SNHG16-miR-506-3p-PTBP1 axis overcomes 5-Fu resistance of GC in vitro and in vivo

**Fig. 9:**
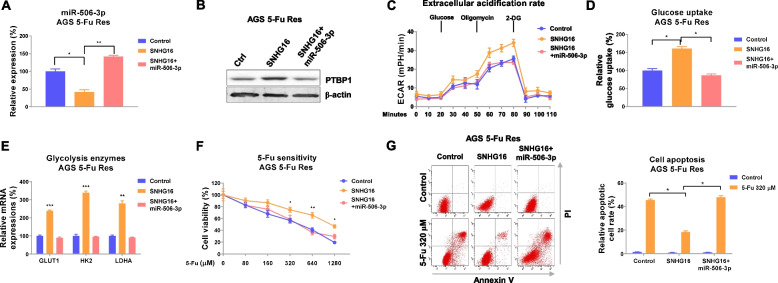
SNHG16 promotes 5-Fu resistance through modulating the miR-506-3p-PTBP1-glycolysis axis. **a**, **b** AGS 5-Fu-resistant cells were transfected with control, SNHG16 alone, or plus miR-506-3p. Expressions of miR-506-3p and PTBP1 were examined by qRT-PCR and Western blot. ***c*** ECAR, ***d*** glucose uptake, and **e** glycolysis enzymes expressions from the above transfected cells were determined. **f** The above transfected cells were treated with 5-Fu at the indicated concentrations, and cell viabilities were determined by MTT and **g** Annexin V apoptosis assay. Data were presented as mean±S.D. *p<0.05; **p<0.01; ***p<0.001

Given that SNHG16 and miR-506-3p-PTBP1-glycolysis inversely modulated 5-Fu sensitivity of gastric cancer, we examined whether SNHG16 accelerated 5-Fu resistance through suppressing the miR-506-3p-PTBP1-glycolysis axis. Thus, AGS 5-Fu-resistant cells were transfected with control, SNHG16 overexpression vector, or SNHG16 plus miR-506-3p. Overexpression of SNHG16 significantly suppressed miR-506-3p expressions, which were further overridden by miR-506-3p restoration (Fig. 9a). Moreover, cells co-transfected with SNHG16 plus miR-506-3p successfully restored PTBP1 expressions (Fig. 9b), suggesting the SNHG16-mediated PTBP1 upregulation was through inhibiting miR-506-3p. Consequently, the ECAR (Fig. 9c), glucose uptake (Fig. 9d), and glycolysis enzymes (Fig. 9e) were effectively rescued by miR-506-3p recovery. Consequently, overexpression of SNHG16 significantly de-sensitized AGS and SGC-7901 5-Fu-resistant gastric cancer cells (Fig. 9f, g, S12). Yet, such de-sensitization was overridden by further miR-506-3p restoration (Fig. 9f, g, S12), suggesting the SNHG16-regulated 5-Fu resistance was through modulating the miR-506-3p-PTBP1-glycolysis axis.

**Fig. 10:**
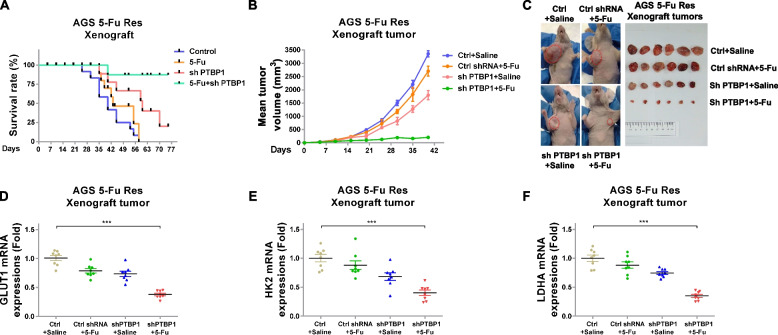
Inhibiting PTBP1 sensitizes gastric tumor to 5-Fu by blocking glycolysis in vivo. **a** Xenograft mice was subcutaneously injected with AGS 5-Fu-resistant cells. Mice were grouped and treated with control, 5-Fu alone, shPTBP1 alone, or the combination of 5-Fu and PTBP1 silencing. The survival rates of mice were recorded. **b** Xenograft tumor volumes from the above treated mice were measured and calculated. **c** Representative mice xenograft tumors from four treatment groups. **d** mRNA expressions of GLUT1, **e** HK2, and **f** LDHA from the above treated xenograft tumors were detected by qRT-PCR. Data were presented as mean±S.D. ***p<0.001

To validate the above proposed in vitro pathway, a xenograft mice model was established. AGS 5-Fu-resistant cells were stably infected with control shRNA or PTBP1 shRNA, followed by subcutaneously injection into the right flank of 6-week-old BALB/c nude mice. After establishing xenograft tumors, mice were treated with 5-Fu or control saline via intraperitoneal injection twice a week. As shown in Fig. 10a, most of the saline treated and control shRNA infected mice who received 5-Fu treatment died within 2 months. Although the PTBP1-knockdown mice without 5-Fu treatment showed slightly increased survival rate, the combination of PTBP1 knockdown and 5-Fu treatment achieved a significantly prolonged survival rate (Fig. 10a). Accordingly, consistent results demonstrated that the mice xenograft tumors underwent PTBP1 silencing plus 5-Fu treatment grew significantly slower than control and 5-Fu alone treatment (Fig. 10b, c). QRT-PCR results from the collected mice tumors showed the mRNA expressions of GLUT1, HK2, and LDHA were apparently suppressed in xenograft tumors with PTBP1 knockdown plus 5-Fu treatment (Fig. 10d, e, and f). Collectively, these results revealed that mice beard xenograft tumors from 5-Fu-resistant, PTBP1-knockdown gastric cancer cells were more sensitive to 5-Fu treatment through inhibition of glycolysis, suggesting the combination of PTBP1 inhibition with 5-Fu treatments renders a synergistically anti-tumor effect against chemoresistant gastric tumors.

## Discussion

Gastric cancer is one of the prevalent malignancies in the world due to its tumorigenesis, development, and metastasis [1, 2]. Although chemotherapeutic approaches have been improved, a large fraction of GC patients developed drug resistance, which limited the effective applications of the 5-Fu-based chemotherapy. This study aimed to investigate the underlying molecular mechanisms and develop effectively anti-chemoresistant strategies against 5-Fu resistance. LncRNAs are known to serve as vital regulators of specific cellular processes [6]. Moreover, lncRNAs are tightly associated with therapeutic options and prognostic values of cancer patients [6]. Here, we observed SNHG16 was positively associated with gastric cancer, consistently with previous reports [9, 10]. Results from this study indicate that SNHG16 is overexpressed in the tumor tissues of GC patients and high expression of SNHG16 is associated with poor prognosis. Furthermore, silencing SNHG16 effectively sensitized 5-Fu-resistant GC cells. Although previous studies have reported that SNHG16 was a gastric cancer cell-favorite molecule [31–33], our results for the first time demonstrated the roles of SNHG16 in 5-Fu-resistant gastric cancer. Given that lncRNAs function as competitive endogenous RNAs (ceRNA) of miRNAs by sponging them to suppress miRNA expressions, leading to recovery of the miRNA target gene expressions, to investigate the molecular mechanisms, we identified miR-506-3p, which has been reported to function as a tumor suppressive miRNA in multiple cancers [28–30], was a directly downstream target of SNHG16 in GC cells by luciferase assay. SNHG16 downregulates miR-506-3p expression through sponging it, suggesting the SNHG16-miR-506-3p ceRNA network could be a promising target for anti-gastric cancer therapy.

Chemoresistant cancer cells exhibit apparently metabolic reprogramming, a phenomenon is known as “Warburg Effect” [13, 14]. Our previous studies have reported that 5-Fu-resistant cancer cells displayed upregulated glycolysis and could be re-sensitized by blocking glycolysis [34]. In this study, we described a SNHG16-miR-506-3p-PTBP1-glycolysis-5-Fu-resistant axis in gastric cancer cells. SNHG16 and PTBP1 were significantly upregulated in 5-Fu-resistant GC cells. In addition, silencing SNHG16 or PTBP1 effectively re-sensitized 5-Fu-resistant cells. Mechanism rescue experiments showed co-transfection of SNHG16 with miR-506-3p effectively overrode the SNHG16-promoted PTBP1, glycolysis, and 5-Fu resistance, suggesting the SNHG16-promoted 5-Fu resistance was through targeting the miR-506-3p-PTBP1-glycolysis axis.

Mounting evidence revealed that PTBP1 functions as a vital regulator for post-transcriptional gene expressions [15–19]. PTBP1 regulates mRNA expressions through messenger RNA (mRNA) splicing, localization, stability, and translation [15–19]. Existing evidence has suggested that PTBP1 upregulated pyruvate kinase 2 (PKM2), a glycolysis key enzyme, leading to a metabolic shift from oxidative phosphorylation to glycolysis in cancer cells [22, 23]. A recent study demonstrated that PTBP1 was upregulated under hypoxia and promoted progressions and chemoresistance of colorectal cancer [35]. In prostate cancer, PTBP1 was known to affect the clinical response to Androgen-deprivation therapy [36], indicting PTBP1 plays vitally regulatory roles in clinical cancer diagnosis and treatment. Moreover, recent studies demonstrated the miR-134/PTBP1 signaling cascade was involved in aerobic glycolysis to enhance osteosarcoma chemoresistance [37], indicating PTBP1 plays important regulatory roles in glucose metabolism. However, the precise molecular targets and underlying mechanisms remain elusive. We highlighted conserved PTBP1 binding motifs in 3′UTRs of major glycolysis enzymes, GLUT1, HK2, and LDHA. The associations between PTBP1 and the 3′UTRs of glycolysis enzymes were validated by RNA pull-down assay and RNA immunoprecipitation assay. Recent study uncovered that PTBP1 stabilizes mRNA by preventing the NMD protein UPF1 from binding 3′UTRs, resulting in evading recognition by the nonsense-mediated mRNA decay pathway [38]. We therefore performed the RNA stability assays and results showed the half-lives of glycolysis enzyme mRNAs were significantly cut down in PTBP1-silencing cells compared with control cells. These results clearly validated that PTBP1 upregulated glycolysis enzyme expressions through direct bind to 3′UTRs of them, resulting in the stabilization of enzyme mRNAs. Furthermore, mice xenograft results strongly supported the above in vitro conclusions. Xenograft tumors underwent PTBP1 silencing plus 5-Fu treatment grew significantly slower than control and 5-Fu alone treatment. Given that PTBP1 functions to regulate mRNA expressions at multiple post-transcriptional levels, the mechanisms underlying the regulation of glycolysis enzymes by PTBP1 are extensive and complex. Thus, other potential mechanisms by which PTBP1 promotes glycolysis are possible. Further investigation is required to address the detailed molecular mechanisms.

## Conclusion

In summary, this study unveiled a promising prospect for the 5-Fu-based therapeutic approaches for GC patients. SNHG16 downregulates miR-506-3p as a ceRNA to de-repress its miRNA target, PTBP1, which upregulates glycolysis key enzymes expressions via binding to the 3′UTRs of enzymes mRNAs, leading to 5-Fu resistance in gastric cancer cells. This study will contribute to the development of new therapeutic strategies against 5-Fu-resistant gastric cancers.

## Supplementary Information

**Additional file 1: Figure S1.** Expressions of SNHG16 and PTBP1 are analyzed from TCGA cancer database on UALCAN.com. **Figure S2.** Quantification of Annexin V cell apoptosis results. **Figure S3.** (A) Expressions of SNHG16 and PTBP1 were detected in SGC-7901 parental and 5-Fu resistant cells. (B) ECAR and (C) glucose uptake were examined in SGC-7901 parental and 5-Fu resistant cells. **Figure S4.** Measurement of the oxygen consumption rate (OCR) from AGS parental and 5-Fu resistant cells. **Figure S5.** Quantification of Annexin V cell apoptosis results. **Figure S6.** Pearson correlation analysis between SNHG16 and glycolysis enzymes from starBase and GEPIA. **Figure S7.** Pearson correlation analysis between PTBP1 and glycolysis enzymes from starBase. **Figure S8.** Effects of stably PTBP1 knockdown on the expressions of glycolysis key enzymes in AGS cells. **, *p* < 0.01; ***, *p* < 0.001. **Figure S9.** The top representatively upregulated and downregulated KEGG enrichment biological processes by shPTBP1. **Figure S10.** Prediction of the binding of PTBP1 on 3’UTRs of glycolysis key enzymes from starBase. **Figure S11.** EMSA experiments to verify the binding capacities of predicted binding motifs. **Figure S12.** Roles of the SNHG16-miR-506-3p axis in 5-Fu sensitivity in SGC-7901 5-Fu resistant cells.

## Data Availability

The datasets used and/or analyzed during the current study are available from the corresponding author on reasonable request.
