# Supplementary material for: Blocking lncRNA-SNHG16 sensitizes gastric cancer cells to 5-Fu through targeting the miR-506-3p-PTBP1-mediated glucose metabolism
Source: Cancer Metab. 2022 Nov 29;10:20. doi: 10.1186/s40170-022-00293-w (PMC9707261; doi:10.1186/s40170-022-00293-w)
Supplement: Supplementary file 1 — Additional file 1: Figure S1. Expressions of SNHG16 and PTBP1 are analyzed from TCGA cancer database on UALCAN.com. Figure S2. Quantification of Annexin V cell apoptosis results. Figure S3. (A) Expressions of SNHG16 and PTBP1 were detected in SGC-7901 parental and 5-Fu resistant cells. (B) ECAR and (C) glucose uptake were examined in SGC-7901 parental and 5-Fu resistant cells. Figure S4. Measurement of the oxygen consumption rate (OCR) from AGS parental and 5-Fu resistant cells. Figure S5. Quantification of Annexin V cell apoptosis results. Figure S6. Pearson correlation analysis between SNHG16 and glycolysis enzymes from starBase and GEPIA. Figure S7. Pearson correlation analysis between PTBP1 and glycolysis enzymes from starBase. Figure S8. Effects of stably PTBP1 knockdown on the expressions of glycolysis key enzymes in AGS cells. **, p < 0.01; ***, p < 0.001. Figure S9. The top representatively upregulated and downregulated KEGG enrichment biological processes by shPTBP1. Figure S10. Prediction of the binding of PTBP1 on 3’UTRs of glycolysis key enzymes from starBase. Figure S11. EMSA experiments to verify the binding capacities of predicted binding motifs. Figure S12. Roles of the SNHG16-miR-506-3p axis in 5-Fu sensitivity in SGC-7901 5-Fu resistant cells. [file 40170_2022_293_MOESM1_ESM.pdf]

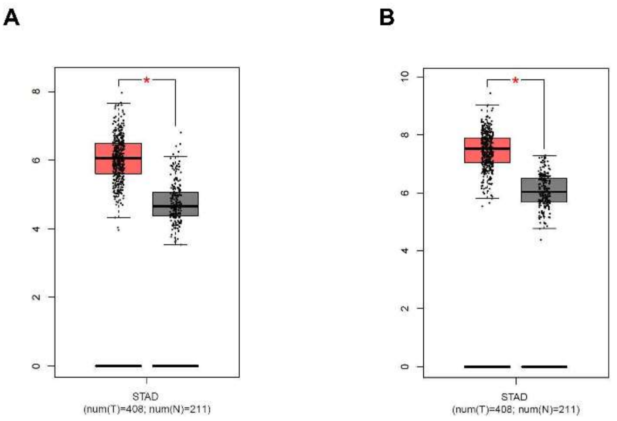

Figure S1. Expressions of SNHG16 and PTBP1 are analyzed from TCGA cancer database on UALCAN.com.

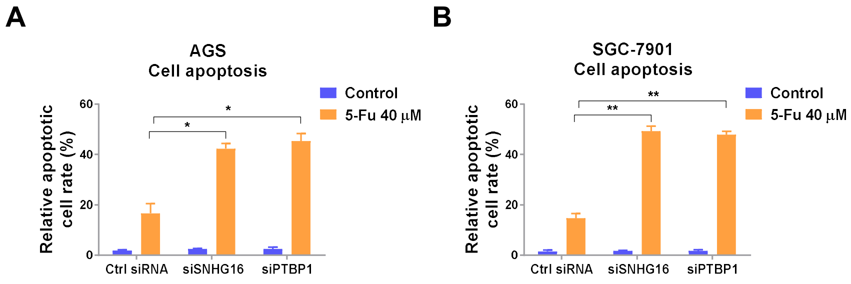

Figure S2. Quantification of Annexin V cell apoptosis results.

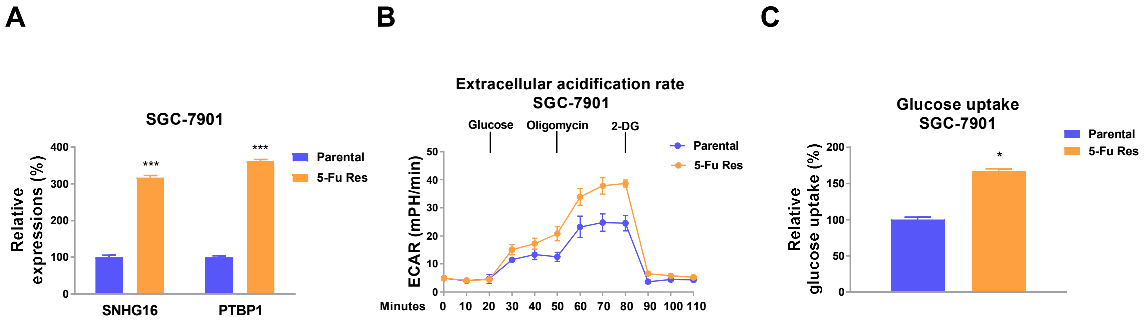

Figure S3. (A) Expressions of SNHG16 and PTBP1 were detected in SGC-7901 parental and 5-Fu resistant cells. (B) ECAR and (C) glucose uptake were examined in SGC-7901 parental and 5-Fu resistant cells.

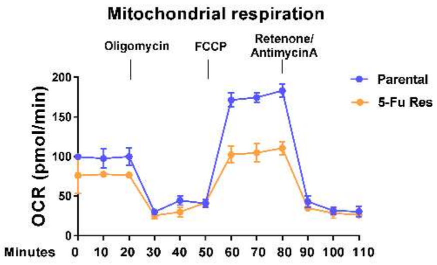

Figure S4. Measurement of the oxygen consumption rate (OCR) from AGS parental and 5-Fu resistant cells.

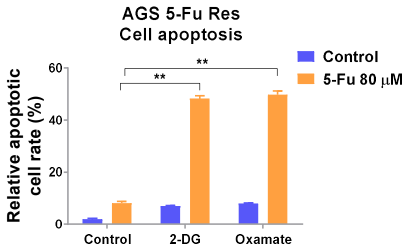

Figure S5. Quantification of Annexin V cell apoptosis results.

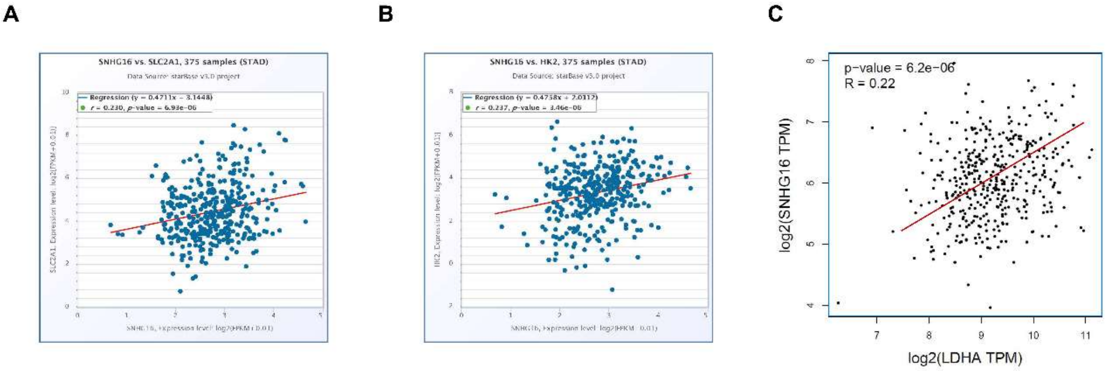

Figure S6. Pearson correlation analysis between SNHG16 and glycolysis enzymes from starBase and GEPIA.

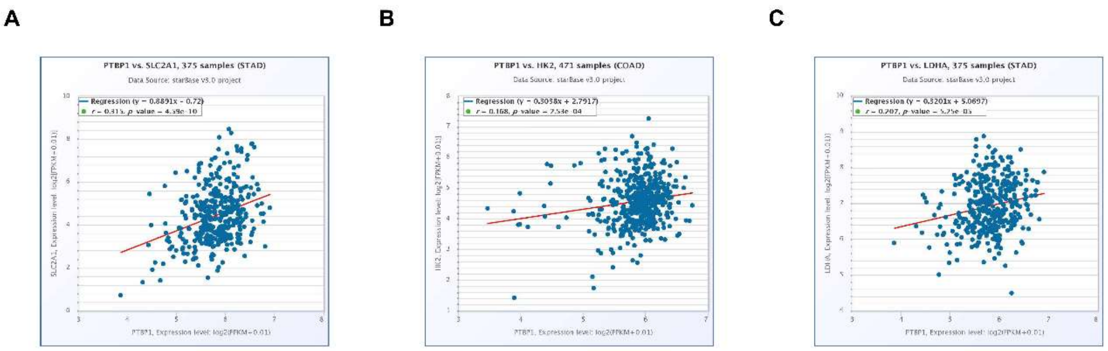

Figure S7. Pearson correlation analysis between PTBP1 and glycolysis enzymes from starBase.

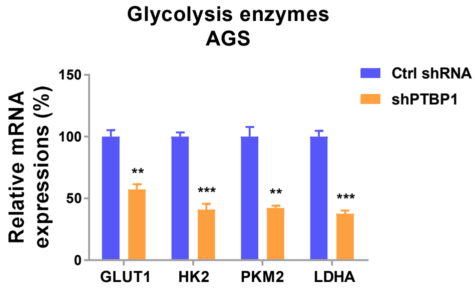

Figure S8. Effects of stably PTBP1 knockdown on the expressions of glycolysis key enzymes in AGS cells. **, $p<0.01$; ***, $p<0.001$.

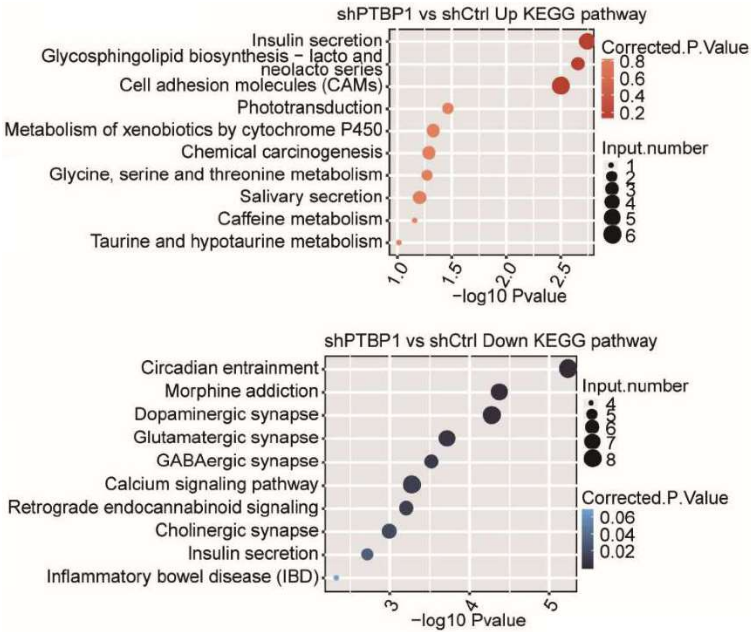

Figure S9. The top representatively upregulated and downregulated KEGG enrichment biological processes by shPTBP1.

A

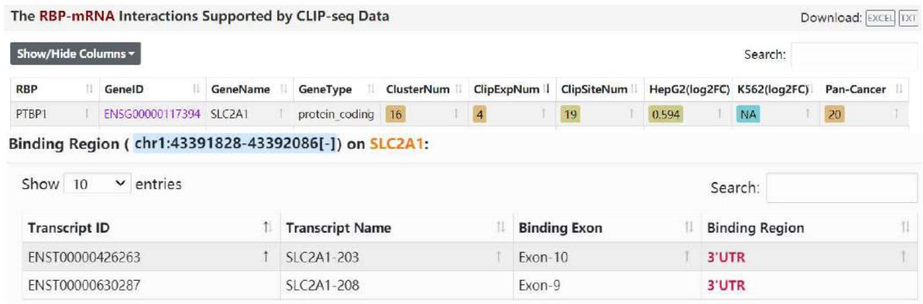

B

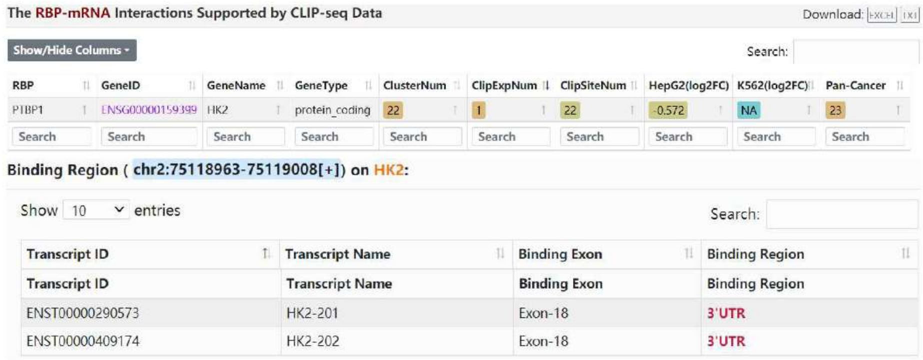

C

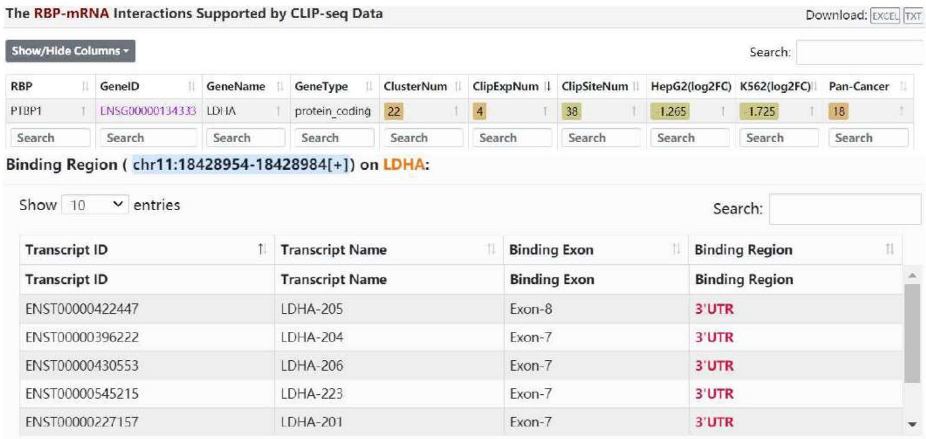

Figure S10. Prediction of the binding of PTBP1 on 3'UTRs of glycolysis key enzymes from starBase.

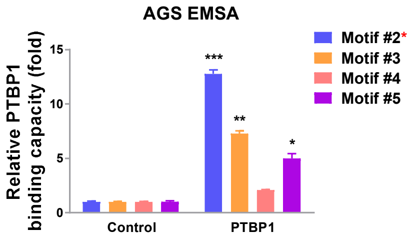

Figure S11. EMSA experiments to verify the binding capacities of predicted binding motifs.

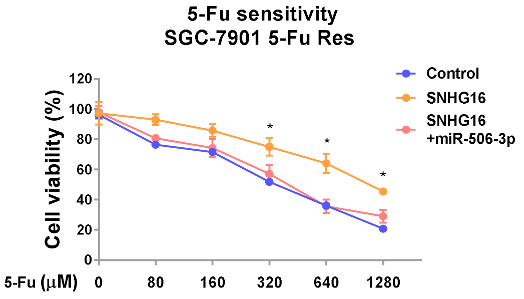

Figure S12. Roles of the SNHG16-miR-506-3p axis in 5-Fu sensitivity in SGC-7901 5-Fu resistant cells.
